# Histone Displacement during Nucleotide Excision Repair

**DOI:** 10.3390/ijms131013322

**Published:** 2012-10-17

**Authors:** Christoffel Dinant, Jiri Bartek, Simon Bekker-Jensen

**Affiliations:** 1 Genome Integrity Unit, Danish Cancer Society Research Centre, Strandboulevarden 49, DK-2100 Copenhagen, Denmark; E-Mail: jb@cancer.dk; 2 Institute of Molecular and Translational Medicine, Faculty of Medicine and Dentistry, Palacky University, Hnevotinska 5, CZ-775 15 Olomouc, Czech Republic; 3 Novo Nordisk Foundation Center for Protein Research, University of Copenhagen, Blegdamsvej 3B, DK-2200 Copenhagen, Denmark

**Keywords:** nucleotide excision repair, histone chaperone, ATP-dependent chromatin remodeling, histone variants

## Abstract

Nucleotide excision repair (NER) is an important DNA repair mechanism required for cellular resistance against UV light and toxic chemicals such as those found in tobacco smoke. In living cells, NER efficiently detects and removes DNA lesions within the large nuclear macromolecular complex called chromatin. The condensed nature of chromatin inhibits many DNA metabolizing activities, including NER. In order to promote efficient repair, detection of a lesion not only has to activate the NER pathway but also chromatin remodeling. In general, such remodeling is thought on the one hand to precede NER, thus allowing repair proteins to efficiently access DNA. On the other hand, after completion of the repair, the chromatin must be returned to its previous undamaged state. Chromatin remodeling can refer to three separate but interconnected processes, histone post-translational modifications, insertion of histone variants and histone displacement (including nucleosome sliding). Here we review current knowledge, and speculate about current unknowns, regarding those chromatin remodeling activities that physically displace histones before, during and after NER.

## 1. Chromatin

In eukaryotes, an octamer of histone proteins H2A, H2B, H3 and H4, two of each, binds to 146 bp-stretches of DNA, forming a nucleosome. Two turns of DNA wrap around the histone octamer and the histone protein H1 binds to the DNA where it extends out of the nucleosome. Repeated units of nucleosomes are referred to as chromatin, and packaging of DNA into chromatin is what allows all eukaryotic DNA, which is ~2 meters long in humans, to fit inside a cell nucleus with a diameter of 10 μm or less. All DNA metabolizing activities, such as transcription, replication, repair and recombination, require direct access to DNA, and such access is restricted by the condensed nature of the chromatin fiber. It follows that DNA repair systems need to overcome these constraints before DNA repair can take place. Subsequently, chromatin is restored, as proposed in the so-called “access, repair, restore” model [1]. In order to allow access to DNA, interactions between histones and DNA are manipulated in a number of ways. Firstly, post-translational modifications (PTMs), mostly of amino acids in the *N*-terminal histone tails but in the case of H2A histones also at the *C*-terminus, are used to regulate higher-order chromatin structure [2]. These include acetylation, methylation, phosphorylation, ubiquitylation and poly-ADP ribosylation [3,4]. Alternatively, nucleosomal structure and interactions through histone tails can be altered by the deposition of histone variants. These variants can typically also be post-translationally modified, as exemplified by the phosphorylation of H2AX’s *C*-terminal residue 139 after DNA damage induction (γ-H2AX). For reviews on histone PTMs in response to DNA damage, see [5,6]. ATP-dependent remodelers and histone chaperones are responsible for a third mechanism that supports DNA repair in the context of chromatin: manipulation of nucleosome composition and occupancy at damaged DNA, and restoration of chromatin upon the completion of repair.

Sliding or removal of histones is performed by SNF2 ATPases. This large protein family is conserved throughout evolution and is a subgroup of the SF2 helicases/translocases, which also include DNA and RNA helicases. The chromatin remodeling activity of the SNF2 domain is probably achieved by active translocation along the DNA, thereby disrupting protein-DNA interactions [7]. ATP-dependent chromatin remodeling complexes exist in four classes: SWI/SNF, INO80, ISWI and CHD. SWI/SNF is associated with nucleosome repositioning and eviction, INO80 with nucleosome sliding and exchange of H2A variants and CHD and ISWI complexes with nucleosome sliding [7,8]. ATP-dependent chromatin remodelers are only able to act on nucleosomes that are already associated with DNA. In order to deposit histones onto DNA, cells require another group of specialized proteins, the histone chaperones. These bind to the positively charged histones, shielding them from negatively charged DNA in order to prevent illegitimate interactions leading to aggregates [9,10]. Instead, histone chaperones promote specific interactions between histones and DNA, which are required for proper nucleosome assembly. Histone chaperones are also involved in chromatin disassembly, but it is unclear at present whether they actively remove histones or act as acceptors for histones that are evicted by ATP-dependent remodelers [2,9,11–13]. It follows that ATP-dependent remodelers have mainly been implicated in the “access” step of NER while histone chaperones are mostly thought to help restore chromatin after repair. There is a large diversity of histone chaperones, based on the presence of protein domains, and the details regarding the mechanisms of histone eviction/deposition are currently unknown. In this review we will focus on the activities of ATP-dependent chromatin remodelers and histone chaperones during nucleotide excision repair (NER).

## 2. Nucleotide Excision Repair

UV-C light, many chemotherapeutic drugs and toxic chemicals in for example cigarette smoke, air pollution or food contaminants are harmful to cells because they induce helix-distorting lesions in DNA. Such distortion causes stalling of both RNA and DNA polymerases and thus constitutes a detrimental obstacle to transcription and replication. In addition, lesions that distort the DNA helix can result in the incorporation of wrong bases during replication, leading to potentially carcinogenic mutations. Cells deal with such DNA damage by activating NER, which excises a ~25 bp stretch of damaged DNA, and subsequently allows resynthesis using the undamaged complementary strand as a template. Mutations in *NER* genes can cause four congenital disorders: *Xeroderma Pigmentosum*, Cockayne syndrome, UV-sensitive syndrome and Trichothiodistrophy, illustrating the importance of this repair pathway in human health [14,15].

Two separate surveillance mechanisms detect DNA lesions and initiate NER: In non-transcribed strands of the genomic DNA (global genome NER or *GG-NER*) XPC-HR23B-CEN2 or DDB2-DDB1-CUL4-RBX1 complexes constitute the recognition modules that detect and mark the site of the lesion (depending on the type of damage). In addition, damage in actively transcribed genes (transcription-coupled NER or *TC-NER*) may be recognized by the lesion-induced stalling of RNA polymerase II, leading to stabilization of its interaction with CSB. Aside from chromatin remodeling promoting transcription elongation, and thereby indirectly lesion recognition upon stalling of RNA polymerase II, there is no indication as of yet that direct damage recognition by DDB2 or XPC is preceded by binding of a remodeling factor. After recognition of DNA damage, *TC-NER* and *GG-NER* are thought to use identical mechanisms to repair DNA. Unwinding of DNA by the two helicase subunits of TFIIH, XPB and XPD, is followed by lesion verification by RPA and XPA, which also aid in correctly aligning the 3′ and 5′ endonucleases XPG and ERCC1-XPF. After incision, DNA is resynthesized using the replication machinery, and the 5′ nick is ligated by Lig I or XRCC1-Lig III. *NER* has been successfully reconstituted *in vitro* on naked as well as chromatinized DNA substrates by adding the above-mentioned *GG-NER* factors [16–19].

If the lesions described above are so abundant that they interfere with the vital process of DNA replication on a global scale, they can elicit a full-blown DNA damage response. This response, which includes signaling by the ATM and ATR kinases to cell cycle checkpoint—apoptosis- and senescence-pathways, is chiefly triggered by the presence of DNA double strand breaks and lesions that massively impede DNA replication [20]. Besides directly impacting on various cell fate pathways, the activated DNA damage response acts to locally shape the chromatin landscape in the vicinity of the DNA lesions, a process that is designed to optimize the repair and restoration of the DNA and the associated chromatin fiber. This chromatin response is initiated by the local phosphorylation of histone H2AX by the ATM and ATR kinases, and the subsequent assembly of two ubiquitin ligase complexes at sites of DNA damage. These sequentially acting complexes, consisting of RNF8, Herc2 and Mdc1 in the first wave and RNF168 in the second wave, act together with Ubc13 to produce non-canonical K63-linked ubiquitin on H2A-type histones [21]. This elaborate and DNA-damage associated chromatin modification is required to recruit downstream components of the DNA damage response, such as 53BP1 and the BRCA1 A complex [22]. ATR is also directly activated by the short stretches of RPA-coated single-stranded DNA that occurs upon processing of NER lesions, which is likely to be accompanied by cell cycle checkpoint signaling. At present, however, it is unclear what the consequences of such signaling would be in a non-replicating cell.

There is growing evidence that different types of DNA damage that require separate pathways for repair, are able to activate the same signaling pathways during the DNA damage response (DDR). For example, phosphorylation of H2AX and the subsequent ubiquitylation of H2A and H2AX occur both at sites of double strand breaks as well as at clusters of NER lesions [23,24]. These histone modifications trigger the recruitment of the same factors in both cases (e.g., BRCA1 and 53BP1). This suggests that some chromatin remodeling activities that are triggered by these DDR histone modifications, rather than a specific type of damage or repair pathway, occur as a general response to genomic insult.

## 3. ATP-Dependent Remodelers

Histone displacement is an important process during the initiation of NER, given that *in vitro* NER on nucleosomal templates is stimulated by remodeling, and a number of chromatin remodeling proteins have been found to be recruited to sites of DNA damage in living cells [5,39]. However, there are only a limited number of studies that have monitored histones in living cells upon DNA damage induction to study their displacement *in situ*. Recently, immobilization of the damage recognition factor DDB2 on a large array of LacO repeats was found to induce large-scale ATP-dependent relaxation of chromatin [40]. In this study, similar results were found when chromatin relaxation was monitored by transient overexpression of GFP-tagged histones. Poly ADP-ribose polymerase (PARP) was required for the relaxation, and PARP and ATP stimulate downstream recognition of the damage by XPC as well, providing evidence that chromatin remodeling promotes DNA repair. None of the SNF2 family members could be shown to be directly involved, but the family members BRG1 and BRM were excluded as being essential for the process. In this regard, it is important to note that DDB2 can more efficiently bind to certain lesions (CPDs) than XPC, and this DDB2-binding likely stimulates damage recognition by XPC [41]. There is evidence that this hand-over from DDB2 to XPC occurs via a direct interaction between the two proteins [42], but chromatin remodeling could be involved as well. The dispensability of BRG1 for DDB2-stimulated XPC recruitment is somewhat surprising because previous studies showed that BRG1 promotes NER and interacts with DDB2 upon UV irradiation [4,25]. Another ATP-dependent remodeler that could be responsible for DDB2-directed chromatin relaxation is CHD4, which has been shown to be recruited to sites of DSBs in a PARP-dependent manner (see also below) [43]. Whether or not DDB2 stimulates NER mainly via a chromatin remodeling process or not remains a topic for further research.

Besides associating with XPC and DDB2 in a UV-dependent manner [4,25], BRG1 was identified as one of a number of SWI/SNF complex subunits to be required for optimal survival of somatic as well as germ cells in *C. elegans* upon UV irradiation [32]. BRG1 was also found to be mutated in several cancer cell lines, including those isolated from breast, prostate, lung, pancreas and colon tumors [44]. Although these data indicate an important function for BRG1 in the DDR, the findings may merely reflect the role of the protein in transcriptional regulation, and no report has of yet shown that BRG1-dependent chromatin remodeling takes place at sites of DNA damage.

Three other subunits of SWI/SNF, Snf5, Snf6 and Swi2, have been implicated in NER by promoting nucleosome rearrangements (Snf6 and Swi2) and impacting on survival upon UV irradiation (Snf5 and Snf6) in *S. cerevisiae* [26,27]. The lack of UV-sensitivity of the *swi2Δ* mutant might reflect a relatively minor role for Swi2p in UV-dependent chromatin remodeling at the locus that was studied (*MFA2* promoter) or possibly redundant functions with other chromatin remodeling factors, such as Snf6 [27].

The above-described ATP-dependent chromatin remodeling activities are most likely involved in the “access” step that promotes binding of repair factors to DNA. Two other SNF2-family complexes have been implicated in NER (INO80 and ISWI), but it is less clear at which step of the repair cascade they function. INO80 appears to have a different function in the UV response in mammals compared to that of yeast. In human colon epithelial cells (HCT116), INO80 binds to DDB1 and is required for the efficient recruitment of XPC and XPA to UV-C induced lesions [28]. Both INO80 itself and one of its binding partners, ARP5, relocalize to damaged areas in the nucleus in a DDB1-dependent manner, suggesting that recruitment of the INO80 complex to UV lesions directly promotes early steps of NER. In *S. cerevisiae* on the other hand, INO80 is recruited to damaged chromatin in a Rad4 (the yeast XPC homolog)-dependent manner and *ino80* mutants are deficient in chromatin restoration after repair, rather than in promoting access prior to repair [29]. Consistent with the above, the role of the INO80 complex also differs in the DSB response between yeast and mammals. In yeast, INO80 recruitment requires DSB-induced phosphorylation of H2A and promotes the removal of nucleosomes, whereas in mammalian cells H2AX phosphorylation is dispensable and, rather, the INO80 subunit ARP5 seems to be required to promote H2AX phosphorylation [45–49]. Thus it seems that mammalian and yeast INO80 complexes have evolved separate functions over time and that the functions of INO80 in yeast cannot be directly extrapolated to higher eukaryotes. A precise function of INO80 during NER is not clear, but the documented function of INO80-class chromatin remodelers, nucleosome sliding and H2A variant exchange, could be relevant during the “access” as well as the “restore” step, or both.

ATPases of the ISWI family exist in several complexes, one of which, ACF, has been implicated in NER. *Drosophila* ACF can stimulate NER *in vitro* on reconstituted di-nucleosomes, containing a lesion in the linker DNA between the nucleosomes [50]. This is probably achieved through a nucleosome sliding mechanism. More recently, ACF1, the second subunit of the ACF complex (besides ISWI), was shown to accumulate at UV-C damaged areas in living human cells [30] and the *C. elegans* ISWI ortholog, ISW-1, was shown to be required for UV resistance, but only at very high doses [32]. These findings indicate that ISWI enzymes are involved in NER, but at present it is not clear at what point of the NER process ISWI exerts its functions. The inherent ability of ISWI to induce nucleosome sliding suggests that the protein may function early in NER, by vacating a stretch of the damaged DNA for binding of repair factors. However, the same sliding activity has previously been associated with chromatin assembly rather than disruption in a number of studies [51–53]. Cells depleted for ACF1 are only slightly sensitive to UV-C [54] yet ACF was shown to be required for efficient G2/M checkpoint activation upon UV-C irradiation [31]. These findings suggest that the ISWI complex ACF may be involved in the “restore” step of NER, but further studies are required to pinpoint a clear function for ISWI in NER.

CHD4 is a member of the fourth class of ATP-dependent remodelers, CHD. There is some indirect evidence that CHD4, part of a larger chromatin-remodeling complex, NuRD, might be involved in NER as well. This is based on the finding that RNF8, immobilized at a LacO repeat array, can recruit CHD4 to chromatin [33]. Different from the typical mechanism of RNF8-dependent recruitment to DNA damage, in this study CHD4 does not recognize ubiquitin conjugates deposited by RNF8, but rather directly interacts with RNF8. It is not clear whether RNF8-dependent recruitment of CHD4 to damaged chromatin also occurs through a direct interaction between the two proteins. In addition, Polo *et al.* show that CHD4 accumulation at sites of DSBs is dependent on PARP activity [43]. Both RNF8-interaction and PAR binding could be responsible for CHD4 accumulation to UV-damaged chromatin as well, but so far the relative contribution of these two binding mechanisms to CHD4 recruitment has not been addressed. The chromatin remodeling activity of CHD4 at DSBs promotes local ubiquitination and RNF168 and BRCA1 recruitment, but it is not required for RNF8 accumulation [55,56]. Together with the finding that CHD4 and RNF8 interact directly, this might indicate that CHD4 functions at a relatively late step during NER, after RNF8 has been recruited, but it does not exclude the possibility that PARP activity recruits CHD4 at an earlier step as well. Until a study directly addresses this issue, the involvement of CHD4 in NER remains speculative.

## 4. Histone Chaperones

Chromatin restoration by ATP-dependent chromatin remodelers requires histone deposition by histone chaperones. CAF-1 was one such chaperone to perform this function in a *Drosophila in vitro* chromatin assembly system [53]. The two histone chaperones Asf1 and CAF-1 have separate but synergistic roles during histone incorporation in response to UV-C irradiation. A current model for the cooperation of Asf1 and CAF-1 predicts that Asf1 binds newly synthesized H3.1/H4 heterodimers and passes them on to CAF-1 that then loads them onto DNA [9,57–61]. In agreement with this, CAF-1 is recruited to sites of UV-C damage, probably via its interaction with PCNA. Asf1, on the other hand, could not be detected on chromatin in Triton-X100 treated cells, indicating that it either interacts with CAF-1 on DNA in a transient manner or that the histone hand-off occurs in the nucleoplasm [35]. Histone deposition by CAF-1 occurs at a late stage after DNA damage induction, probably upon the successful completion of NER [34].

An important question is whether epigenetic information manifested by post-translational modifications on histone tails is accurately maintained after histone removal and replacement upon DNA repair. During normal replication, epigenetic inheritance is probably ensured by the direct transfer of parental histones to both strands of newly synthesized DNA behind the fork [62,63]. The remaining space on the DNA is filled by new histones that, in the case of H3 and H4, are acetylated on specific residues (H3K56Ac and H4K91Ac) before incorporation [59,64]. PTMs on the parental histone tails could then function as a blueprint for the modification of new histones, thereby restoring the epigenetic information. It appears that, as is the case during replication, naive H3 histones are acetylated on lysine 56 prior to incorporation into repaired chromatin [65–67]. The presence of this modification is required to signal the completion of repair in order to switch off the DNA damage checkpoint. In agreement with this, an earlier study found that upon completion of NER, restored chromatin contained new H3 molecules that had not previously been incorporated into chromatin [34]. This implies that any epigenetic marks that existed before histone removal from damaged chromatin are reestablished *in situ*, if at all, most likely by copying PTMs on surrounding histones.

The histone chaperone FACT (hSPT16 and SSRP1 in human, Spt16, Pob3 and Nhp6 in yeast) was initially identified as a factor that promotes transcription through nucleosomal templates [68,69]. The enhancement of DNA accessibility by FACT is probably achieved by interruption of the H3-H4 tetramer/H2A-H2B dimer interface. This interruption can result in eviction of H2A-H2B dimers, but it is not clear at the moment whether dimer eviction is essential for the function or if increased access to DNA is already achieved by the initial reorganization of the nucleosome by FACT [12,70].

There are various indications that FACT is involved in the DDR. First of all, CK2, an important and UV-induced kinase, and FACT are found together in a complex that specifically phosphorylates p53 on serine 392 upon UV irradiation [37,71]. Ser-392 phosphorylation of p53, which activates this important transcription factor, occurs exclusively upon UV, and not other types of damage, and FACT appears to be required for CK2 to selectively target this residue. FACT can also bind directly to lesions on damaged DNA (cisplatin-induced crosslinks and UV-C damage) via the HMG-1 domain of SSRP1 [72,73]. In the absence of hSPT16, this binding to damaged DNA is strongly inhibited, suggesting that the hSPT16-SSRP1 interaction promotes a confirmation that exposes the HMG-1 domain to DNA [73]. The available data on FACT interaction with chromatin in response to DNA damage are at present incomplete. First, one study shows that CK2-dependent phosphorylation of SSRP1 enhances FACT binding to UV-damaged DNA [72], while a different paper reports that this modification inhibits binding of FACT to non-modified linear DNA [74]. Second, FACT has been found both to accumulate at sites of DNA damage [72,75] and to be removed from damaged chromatin [76]. While these conflicting results may be attributed to the use of different DNA substrates, they significantly complicate the interpretation of, in particular *in vitro*, data on the potential activities of FACT during the DDR.

In contrast to the histone chaperones CAF-1 and Asf1, FACT has not been studied specifically within the context of NER. However, from a number of studies on the mechanistics of FACT-directed histone exchange, we may try to extrapolate the functions of FACT in UV-damaged cells. Measuring exchange of H2A-H2B dimers for H2AX-H2B, and *vice versa*, in an *in vitro* assay with reconstituted nucleosomes, FACT was shown to catalyze both reactions to the same extent [77]. Conversely, DNA-PK-phosphorylated H2AX-H2B dimers were preferentially removed from nucleosomes and replaced with H2A-H2B. The physiological relevance of this increased exchange of γ-H2AX is difficult to determine, since an earlier *in vivo* H2AX mobility study found no indication of enhanced removal of phosphorylated H2AX from chromatin after DNA double strand break induction [78]. In addition, as FACT can catalyze both insertion and eviction of H2A-H2B and H2AX-H2B dimers, it is likely that free histone supply will play a regulatory role in determining which histone variants are incorporated and which are removed *in vivo*. FACT activity can also be regulated via poly-ADP-ribosylation of SPT16 by PARP1, which causes FACT to dissociate from chromatin and represses histone exchange [76,77]. This is somewhat contradictory to the established functions of PARP1, since it suggests that PAR-sylation of SPT16 inhibits the chromatin accessibility of FACT, while at the same time PARP1 activity was found to play a role in early chromatin relaxation in response to UV-C irradiation [40]. Although there is no indication from Luijsterburg *et al*. that FACT is involved in this early chromatin relaxation, FACT is recruited to UV lesions and other types of DNA damage [72,79], challenging the idea that SPT16 PAR-sylation plays a role early during DNA repair. It is possible that PARP1-induced dissociation of FACT from chromatin upon genotoxic stress occurs mainly in areas where FACT actively promotes transcription, while still being able to bind damaged DNA directly. In agreement with this, DNA damage causes the release of SSRP1 from nucleoli, where it is thought to be involved in RNA Polymerase I-directed transcription, and lack of SSRP1 relocalization, caused by DNA-PK inhibition, correlates with a cisplatin sensitivity that is similar to that observed after RNAi-mediated knockdown of SSRP1 [80].

FACT is able to promote both the eviction and insertion of H2A-H2B dimers, which suggests that it could function both at the “access” and at the “restore” steps of NER. In light of the fact that it promotes DNA access during transcription and replication, however, it is likely that FACT is activated early upon damage induction, probably through direct binding to the lesion or via histone interactions, to promote repair.

NPM1 is a histone chaperone that belongs to the nucleoplasmin/nucleophosmin (NPM) family, which has been proposed to function in many diverse processes, including ribosome biogenesis, cell cycle regulation, mRNA splicing, apoptosis, centrosome replication and genome stability [81]. The founding member of the NPM family, nucleoplasmin/NPM2, was the first histone chaperone to be identified in a nucleosome assembly assay performed on Xenopus extracts [82]. NPM1 had already been identified by then [83], but it was only much later that the protein was shown to possess histone chaperone activity [84,85]. Involvement of NPM1 in NER was first suggested when it was found that its overexpression increased NER activity and conferred resistance against UV-induced cell death [38]. Whether this relates to a direct involvement of NPM1 in NER, or indirect via upregulation of transcription of essential NER genes, could not be concluded from this study. A phosphorylated form of NPM1 was recently reported to be recruited to RNF8-dependent ubiquitin conjugates upon double strand break induction, indicating a direct involvement in the DDR [86]. This suggests that NPM1 could also be recruited to UV lesions, although this needs to be confirmed by direct experiments. Interestingly, the finding that NPM1 knockdown does not induce sensitivity to IR whereas it increases UV-induced cell killing, suggests that it is more important for NER than for DSB repair [38,86]. At the moment there is a lack of mechanistic data on NPM1 activity during the DDR to pinpoint a function for this histone chaperone. Data on transcription regulation by NPM1 indicate that it enhances DNA accessibility within chromatin suggesting a function in the “access” step of NER [85]. On the other hand, RNF8 acts at a late stage in the NER cascade, requiring single-stranded DNA repair intermediates [23], which would suggest that ubiquitin-mediated recruitment of NPM1 occurs during the “restore” rather than the “access” step of NER. Clearly, more studies are required to shed light on this issue.

## 5. Conclusions

In response to DSBs, large stretches of chromatin are modified by ubiquitination and phosphorylation of histones and other proteins, resulting in the formation of large multiprotein structures within nuclei, termed ionizing radiation-induced foci (IRIF). These large-scale chromatin modifications are essential not just for checkpoint signaling, but also for efficient repair. NER activation does not lead to the formation of such discrete and microscopically discernible structures, indicating that large-scale chromatin modifications are not an important part of the UV-induced DDR. In addition, the chromatin modifications that occur during NER only appear after repair is well under way. This suggests that activation of the RNF8 cascade upon UV damage might not serve the same purpose as it does in response to DSB formation. In fact, even though a large part of the DDR involving chromatin signaling through post-translational modifications is shared amongst different DNA repair pathways, it is not known whether all downstream events, e.g., recruitment of 53BP1, BRCA1 and some chromatin remodelers, are essential for all these repair pathways to the same extent. It is possible that some chromatin remodeling activities triggered by local chromatin PTMs are important for efficient DSB repair, but have a negligible effect on NER efficiency.

For most of the remodelers discussed in this review there exists insufficient data to determine an exact function during NER. Often we cannot yet be sure whether their contribution to DNA repair involves remodeling activity *per se* because many chromatin remodelers have other functions aside from histone displacement. In order to gain better insight into the mechanisms of action of chromatin remodelers in response to genotoxic insults, specialized techniques have to be employed and new assays have to be developed to study the dynamics of histones in living cells during DNA repair. In conjunction with studies of genetic interdependencies and *in vitro* chromatin remodeling assays, such assays should help us elucidate the intricacies of chromatin remodeling during NER. Finally, given the rapidly expanding spectrum of genetic aberrations affecting the function of various components of NER, DNA damage signaling and chromatin remodeling pathways [87], current research in this area of biomedicine is expected to provide novel insights valuable for improved diagnosis and treatment of life-threatening disorders such as neurodegeneration, premature aging and cancer.

## Figures and Tables

**Figure 1:**
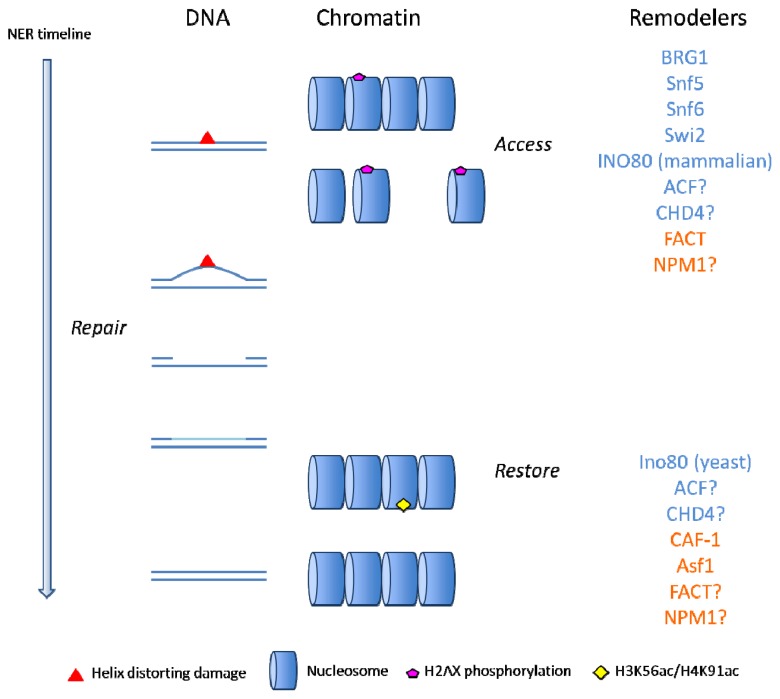
Schematic representation of the “access, repair, restore” model for nucleotide excision repair (NER). For simplification purposes, DNA and chromatin (without DNA) are depicted separately. On the right, the remodeling proteins are listed at either the “access” or “restore” step. ATP-dependent chromatin remodelers are shown in blue and histone chaperones in red.

**Table 1:** ATP-dependent remodelers and histone chaperones implicated in NER and their documented activity.

Remodeler	Activity during NER	Species	Reference
*ATP-dependent remodelers*			

BRG1	Interacts with XPC and DDB2	Mammalian	[4,25]
	Required for UV survival (extent not determined)		

Snf5	Required for UV survival	Yeast	[26]

Snf6	Required for UV survival	Yeast	[26,27]
	Promotes nucleosome rearrangements in response to UV		
Swi2	Promotes nucleosome rearrangements in response to UV	Yeast	[27]

INO80	Binds DDB1 and is required for recruitment of XPC and XPA	Mammalian	[28]
	Recruited to UV damage by Rad4 and involved in chromatin restoration after repair	Yeast	[29]
ACF1	Recruited to UV damage	Mammalian	[30,31]
	Required for G2/M checkpoint activation upon UV irradiation		

ISW1	Required for UV survival at high doses	*C. elegans*	[32]

CHD4	Directly interacts with RNF8	Mammalian	[33]

*Histone chaperones*			

CAF-1	Loads newly-synthesized H3-H4 dimers onto chromatin after NER	Mammalian	[34]

Asf1	Transfers H3K56Ac-H4 tetramers to CAF-1 for post-repair deposition	Mammalian	[35]
	Required for UV survival (extent not determined)	Yeast	[36]

FACT	Directs CK2-dependent p53 phosphorylation upon UV damage	Mammalian	[37]

NPM1	Overexpression promotes UV survival	Mammalian	[38]
